# Microscopic origins of the large piezoelectricity of leadfree (Ba,Ca)(Zr,Ti)O_3_

**DOI:** 10.1038/ncomms15944

**Published:** 2017-06-20

**Authors:** Yousra Nahas, Alireza Akbarzadeh, Sergei Prokhorenko, Sergey Prosandeev, Raymond Walter, Igor Kornev, Jorge Íñiguez, L. Bellaiche

**Affiliations:** 1 Physics Department and Institute for Nanoscience and Engineering, University of Arkansas, Fayetteville, Arkansas 72701, USA; 2 Research Institute of Physics, Southern Federal University, Rostov on Don 344090, Russia; 3 Laboratoire Structures, Propriétés et Modélisation des Solides, Université Paris-Saclay, CentraleSupélec, CNRS-UMR8580, Grande Voie des Vignes, 92295 Châtenay-Malabry Cedex, France; 4 Department of Materials Research and Technology, Luxembourg Institute of Science and Technology, (LIST), 5 avenue des Hauts-Fourneaux, L-4362 Esch/Alzette, Luxembourg

## Abstract

In light of directives around the world to eliminate toxic materials in various technologies, finding lead-free materials with high piezoelectric responses constitutes an important current scientific goal. As such, the recent discovery of a large electromechanical conversion near room temperature in (1−*x*)Ba(Zr_0.2_Ti_0.8_)O_3_−*x*(Ba_0.7_Ca_0.3_)TiO_3_ compounds has directed attention to understanding its origin. Here, we report the development of a large-scale atomistic scheme providing a microscopic insight into this technologically promising material. We find that its high piezoelectricity originates from the existence of large fluctuations of polarization in the orthorhombic state arising from the combination of a flat free-energy landscape, a fragmented local structure, and the narrow temperature window around room temperature at which this orthorhombic phase is the equilibrium state. In addition to deepening the current knowledge on piezoelectricity, these findings have the potential to guide the design of other lead-free materials with large electromechanical responses.

## Abstract (web summary)

(Ba,Ca)(Zr,Ti)O_3_ ceramics have attracted interest as lead-free materials with large piezoelectric responses. Here, the authors use an atomistic model to show the electromechanical properties are due to a complex microstructure and polarization fluctuations in the narrowly stable orthorhombic phase.

Piezoelectricity is a physical phenomenon that converts mechanical into electrical energy and vice-versa (see ref. 1 and references therein). It has been used for devices such as sensors, actuators or ultrasonic motors^2^,^3^. Up to now, the materials exhibiting the highest piezoelectric responses include lead ions, such as Pb(Zr,Ti)O_3_ or Pb(Mg,Nb,Ti)O_3_ (refs 4, 5, 6, 7, 8), that introduce toxicity concerns. As a result, the search for lead-free compounds exhibiting large room-temperature piezoelectricity constitutes an important current research direction that is partially driven by regulations announced by several countries^9^,^10^. In that regard, the discovery of a large electromechanical response found at room temperature in the (1−*x*)Ba(Zr_0.2_Ti_0.8_)O_3_−*x*(Ba_0.7_Ca_0.3_)TiO_3_ solid solutions with *x*=0.50 (to be denoted as BCTZ-0.5 in the following) and reported in ref. 11 is a major finding.

Interestingly, the microscopic origin of such large piezoelectricity in this lead-free system remains subject to debate. For instance, the experimental study of ref. 11 suggests that it arises from the proximity of a tricritical point, where two ferroelectric phases of rhombohedral and tetragonal symmetries meet with the paraelectric phase of cubic symmetry. In contrast, the combined theoretical and experimental investigation of ref. 12 proposes that the highest piezoelectric coefficients are reached at the boundary between ferroelectric phases of orthorhombic and tetragonal symmetries as a result of a combination of reduced anisotropy energy, high polarization and enhanced elastic softening. The experimental analyses of refs 13, 14 offer yet another explanation, pointing out the coexistence of tetragonal, orthorhombic and rhombohedral phases and the strong electric-field-dependency of their relative contributions to the total system as the culprits responsible for the large observed electromechanical response. On the basis of the polarization-rotation mechanism proposed in lead-based materials^4^,^15^,^16^,^17^,^18^, it is also legitimate to wonder if an overlooked low-symmetry phase, inside which the spontaneous polarization easily rotates, may be responsible for large piezoelectricity in (Ba_0.85_Ca_0.15_)(Zr_0.10_Ti_0.90_)O_3_.

A plausible explanation for the paucity of knowledge of BCTZ-0.5 is that atomistic simulations, which have been particularly important to understand piezoelectricity in lead-based materials^15^,^16^,^17^,^18^, are currently lacking for (1−*x*)Ba(Zr_0.2_Ti_0.8_)O_3_−*x*(Ba_0.7_Ca_0.3_)TiO_3_ compounds. This lack of simulations is likely due to the difficulty in realistically mimicking these latter solid solutions, since not only do they possess chemical mixing at both the A and B sublattices of the ABO_3_ perovskite structure, but can also exhibit local inhomogeneities (especially if phase coexistence occurs as advocated in refs 13, 14). As a result, large supercells are most likely required to accurately model BCTZ-0.5, which is typically problematic from the standpoint of memory and computational time.

Here, we build a large-scale atomistic approach to tackle room-temperature piezoelectricity in (Ba_0.85_Ca_0.15_)(Zr_0.10_Ti_0.90_)O_3_. The use of such a scheme leads to a successful explanation of its origin that we find residing in the dyadic combination of the narrow temperature range of stability of the macroscopic orthorhombic phase near 300 K, and the flatness of the free energy associated with this orthorhombic phase, which allows large fluctuations of the polarization around its equilibrium value. Such macroscopic effects are also associated with specific characteristics of the local structures, including the existence of the so-called percolating cluster (which is of orthorhombic symmetry) while its strength (that is, volume per cent occupied in the material) is found here to directly correlate with piezoelectricity. It is also worth realizing that clusters of orthorhombic symmetry are not ingredients of the widely used Comes–Guinier–Lambert^19^, which implies that this model ought to be generalized to be more realistic.

## Results

### Atomistic scheme

We adopt the virtual crystal approximation (VCA)^20^,^21^ mimic (1−*x*)Ba(Zr_0.2_Ti_0.8_)O_3_−*x*(Ba_0.7_Ca_0.3_)TiO_3_ with *x*=0.50. We first model a virtual 〈A〉〈B〉O_3_ simple perovskite system, for which the 〈A〉 atom involves a compositional average of Ba and Ca potentials of 85 and 15% respective contributions, while the 〈B〉 atom is built from a mixing of the Zr and Ti potentials of 10 and 90% respective contributions. An effective Hamiltonian (H_eff_) is then developed for this 〈A〉〈B〉O_3_ system. As in ref. 22, the degrees of freedom of this H_eff_ are, for each 5-atom unit cell *i*, the local soft mode **u**_*i*_ that is proportional to the electric dipole moment of the cell, the *η*_H_ homogeneous strain tensor, and inhomogeneous-strain-related dimensionless displacements {**v**_*i*_}. Technically, the various {**u**_*i*_} and {**v**_*i*_} are, respectively, centred on the 〈B〉 and 〈A〉 sites. The analytical expression for the total internal energy of this effective Hamiltonian is the one provided in ref. 22 for pure BaTiO_3_, and therefore contains a local-mode self-energy, a long-range dipole–dipole interaction, a short-range interaction between soft modes, an elastic energy, and an interaction between the local modes and local strains. In particular, the local-mode self-energy is given by:


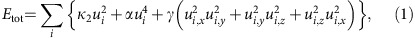


where the sum runs over all the 〈B〉 sites and where (*u*_*i*,*x*_, *u*_*i*,*y*_, *u*_*i*,*z*_) are the Cartesian components of **u**_*i*_ in the orthonormal basis formed by the [100], [010] and [001] pseudo-cubic directions. In the first step, the parameters *κ*_2_, *α* and *γ* are determined, along with the other coefficients of the effective Hamiltonian, by performing density functional theory calculations within the VCA approach^21^ on small 〈A〉〈B〉O_3_ cells (less than 20 atoms). In a second step, Monte-Carlo (MC) simulations using *E*_tot_ are conducted on large supercells (typically of 12 × 12 × 12 or 18 × 18 × 18 dimensions) made of 〈A〉〈B〉O_3_. During these simulations, *κ*_2_ is varied to fit the experimental value of the Curie temperature^11^,^12^,^23^ (since H_eff_ techniques can underestimate the paraelectric–ferroelectric transition temperature^22^,^24^) and *γ* is slightly adjusted to reproduce the measured lowest transition temperature observed in refs 12, 23 for (1−*x*)Ba(Zr_0.2_Ti_0.8_)O_3_−*x*(Ba_0.7_Ca_0.3_)TiO_3_ solid solutions having *x*=0.50. For comparison, we also computed finite-temperature properties of pure BaTiO_3_ (BTO), as arising from the use of the effective Hamiltonian of ref. 24.

### Phase transitions

**Figure 1:**
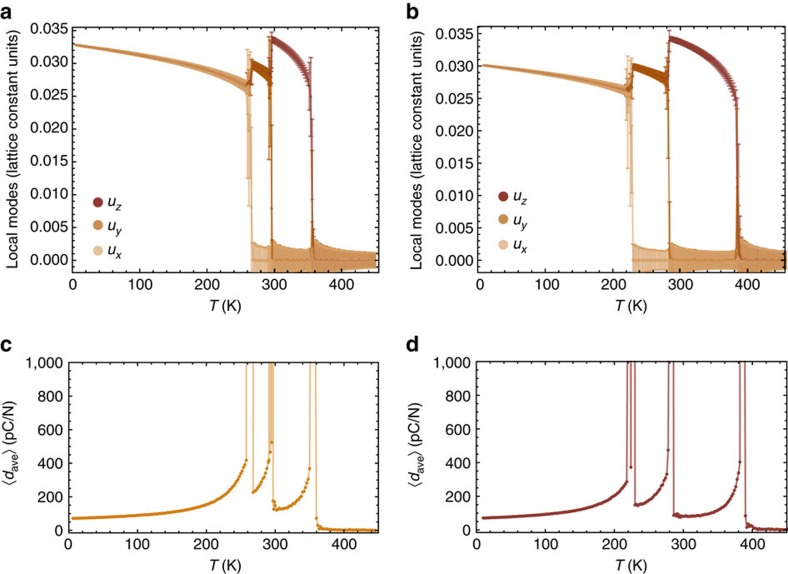
Temperature dependence of macroscopic properties. (**a**,**b**) Report the evolution with temperature *T* of the supercell average of the local modes (in lattice constant units) of (1−*x*)Ba(Zr_0.2_Ti_0.8_)O_3_−*x*(Ba_0.7_Ca_0.3_)TiO_3_ with *x*=0.50 (BCTZ-0.5) and BaTiO_3_ (BTO), respectively. Error bars indicate s.d., and are less than or equal to the size of the points when not visible. (**c**,**d**) Show the evolution with temperature *T* of the average 〈*d*_ave_〉 piezoelectric coefficient (see text) for BCTZ-0.5 and BTO, respectively. Note that 〈*d*_ave_〉 reaches values larger than 1,000 at the *Pm*
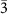
*m*−*P*4*mm*, *P*4*mm*−*Amm*2 and *Amm*2−*R*3*m* transitions. Such values are not shown here because they will make it challenging to see piezoelectricity of the order of hundreds of pC/N in **c**,**d**. Results in all panels are obtained from the use of 18 × 18 × 18 supercells.

The results of these MC simulations for BCTZ-0.5 and BTO are shown in Fig. 1a,b for the Cartesian components of the supercell average of the local modes, 〈**u**〉 (which is directly proportional to the spontaneous polarization), when averaging over 4 million MC sweeps and using 18 × 18 × 18 supercells. The *x*, *y* and *z* axes are chosen along the [100], [010] and [001] pseudo-cubic directions, respectively. The computations for BCTZ-0.5 correctly qualitatively and even quantitatively reproduce the three observed successive (first-order) transitions^12^,^23^ when cooling down the system: first, a paraelectric cubic *Pm*
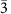
*m* to ferroelectric tetragonal *P*4*mm* transition at around 360 K, for which the *z* component of 〈**u**〉 becomes nonzero; second, a *P*4*mm* to ferroelectric orthorhombic *Amm*2 transition near 297 K (that is, very close to room temperature), for which the *y* component of the supercell average of the local modes suddenly becomes nonzero and equal to the *z* component; and third, a *Amm*2 to ferroelectric rhombohedral *R*3*m* transition at ≃270 K, for which all the Cartesian components of 〈**u**〉 are now nonzero and equal to each other. In Supplementary Note 2, it is also demsonstrated that the effective Hamiltonian scheme used within the VCA approach (with a rescaling of the *κ*_2_ and *γ* parameters) can correctly reproduce the temperature–compositional phase diagram of (1−*x*)Ba(Zr_0.2_Ti_0.8_)O_3_−*x*(Ba_0.7_Ca_0.3_)TiO_3_ (BCTZ-*x*) for *x* ranging between 0.25 and 0.65, as well as other properties of BCTZ-*x*, which further attests to the validity of the presently used numerical method. Moreover, for pure BaTiO_3_, Fig. 1b shows that the *Pm*
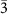
*m*−*P*4*mm*, *P*4*mm*−*Amm*2 and *Amm*2−*R*3*m* transitions are predicted to be about 384, 283 and 226 K, which agree reasonably well with the corresponding experimental values of 400, 280 and 180 K (refs 25, 26).

### Piezoelectricity

Piezoelectric coefficients, *d*_*ij*_, of BCTZ-0.5 and BTO are calculated using the correlation-function approach of ref. 27, that is:


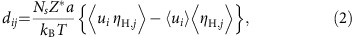


where *T* is the temperature, *k*_B_ the Boltzmann constant, *N*_*s*_ the total number of 5-atom cells composing the supercell, Z* the Born effective charge associated with the soft mode and *a* the 5-atom lattice constant. *u*_*i*_ is the *i*-component of the supercell average of the local mode at a given MC sweep, and *η*_H,*j*_ is the *j* component of the homogeneous strain tensor (in Voigt notation) at this MC sweep. The 〈〉 symbol denotes statistical averages over the different MC sweeps. Figure 1c,d report an averaged computed piezoelectric coefficient, 〈*d*_ave_〉, for BCTZ-0.5 and BaTiO_3_, respectively. More precisely, 〈*d*_ave_〉 is equal to 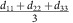 (for the aforementioned (*x*, *y*, *z*) basis) in the R3m phase, since *d*_11_, *d*_22_ and *d*_33_ coefficients are all nonzero and equal to each other in this phase. On the other hand, 〈*d*_ave_〉 is chosen to be 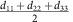 (respectively, *d*_11_+*d*_22_+*d*_33_) in the *Amm*2 (*P*4*mm*) phase because only two (one) of these three coefficients are (is) nonzero there. We also practically choose 〈*d*_ave_〉 to be equal to *d*_11_+*d*_22_+*d*_33_ in the paraelectric phase, as in the tetragonal ferroelectric state (note that all three aforementioned choices provide the same 〈*d*_ave_〉 in the *Pm*
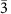
*m* phase since it has no piezoelectricity).

In BCTZ-0.5, large values of this piezoelectric coefficient exist in the temperature range associated with the stability of the *Amm*2 phase, as consistent with the experimental findings of refs 11, 12. In particular, the computed averaged 〈*d*_ave_〉 coefficient is always bigger than ≃225 pC/N and can be as high as 525 pC/N in the macroscopic *Amm*2 phase of BCTZ-0.5 (note that piezoelectric coefficients larger than 525 pC/N shown in Fig. 1c correspond to frequent fluctuations between different macroscopic phases, such as *Amm*2 and *P*4*mm*, and are therefore inherently linked to phase transitions). In contrast, the piezoelectric coefficient can be as small as ≃150 pC/N and does not exceed values of about ≃330 pc/N in the macroscopic *Amm*2 state of BaTiO_3_. Interestingly and unlike in lead-based Pb(Zr,Ti)O_3_ and Pb(Mg,Nb,Ti)O_3_ solid solutions near their morphotropic phase boundary^4^,^15^,^16^, these large piezoelectric responses in BCTZ-0.5 are not due to the existence of a low-symmetry (that is, monoclinic) phase that is associated with the ease of rotating the polarization, since our calculations reported in Fig. 1a (as well as corresponding data related to strain tensors that are not shown here) indicate that they occur within a macroscopic orthorhombic phase.

It is also worthwhile to realize that Fig. 1c further predicts that BCTZ-0.5 should also have large piezoelectric coefficients (larger than 200 pC/N) in the rhombohedral R3m phase in the vicinity of the *Amm*2–*R*3*m* phase transition, namely for temperature varying between 260 and 266 K, as well as close to the Curie temperature of ≃360 K, which is consistent with the experimental data of ref. 12.

### Fluctuations

**Figure 2:**
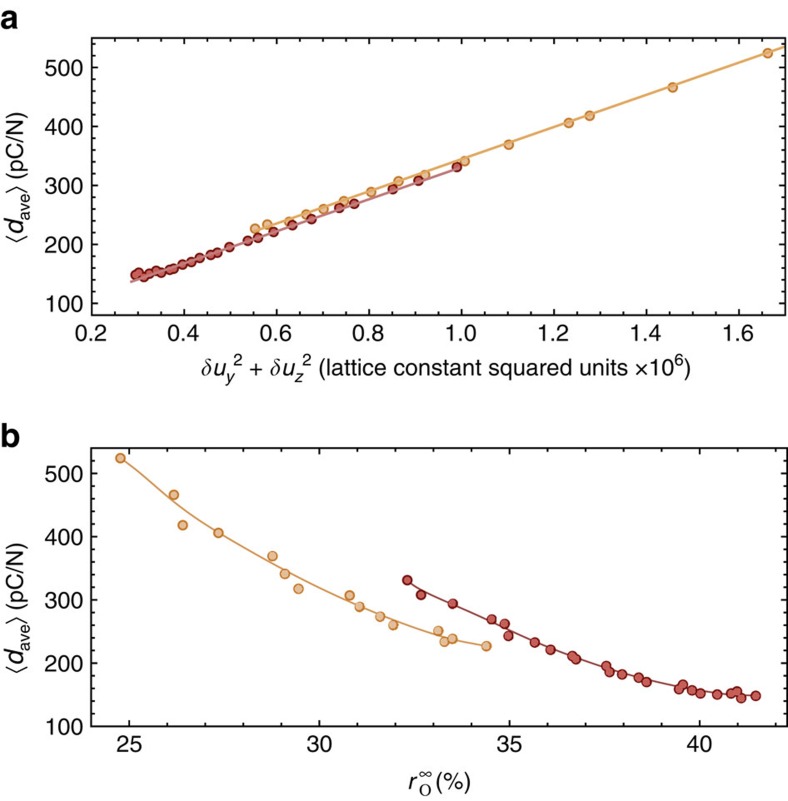
Dependence of piezoelectricity on fluctuations and percolation. (**a**) Reports the dependency of the average 〈*d*_ave_〉 piezoelectric coefficient (see text) on fluctuations of the polarization in the macroscopic *Amm*2 phase of (1−*x*)Ba(Zr_0.2_Ti_0.8_)O_3_−*x*(Ba_0.7_Ca_0.3_)TiO_3_ with *x*=0.50 (BCTZ-0.5) in yellow symbols, and BaTiO_3_ (BTO) in red symbols. *δu*_*y*_ and *δu*_*z*_ are the error bars of the *y* and *z* component of the supercell average of the local mode, respectively, displayed in Fig. 1a,b. (**b**) Shows the dependency of the average 〈*d*_ave_〉 piezoelectric coefficient on the 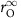 strength of the percolating O cluster (that is, the infinite cluster that spreads from one side of the supercell to its opposite side along the [100], [010] or [001] pseudo-cubic directions^36^) in the macroscopic *Amm*2 phase of BCTZ-0.5 (yellow symbols) and BTO (red symbols). Results correspond to the use of a 18 × 18 × 18 supercell. Solid lines are linear least-square fits in **a**, and guide for the eyes in **b**.

To better understand the piezoelectric responses within the *Amm*2 phases of BCTZ-0.5 and BTO, as well as their differences and origins, Fig. 2a reports 〈*d*_ave_〉 versus 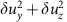 in the ferroelectric orthorhombic state of these two materials, where *δu*_*y*_ and *δu*_*z*_ are the error bars that are associated with the *y* and *z* components of the supercell average of the local mode that are shown in Fig. 1a,b. These error bars quantify the fluctuation of the polarization about its macroscopic average, which is oriented along the [011] direction. What is remarkable is that not only 〈*d*_ave_〉 is found to nearly linearly increase with 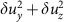, but also that this linear relationship is rather similar between BCTZ-0.5 and BaTiO_3_ (Supplementary Note 3 provides an understanding of the linear relationship between 〈*d*_ave_〉 and 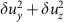. We did not include *δu*_*x*_ in the computation of the fluctuations reported in the horizontal axis of Fig. 2a because the polarization in the orthorhombic phase has a vanishing *x* component). One can thus assert that BCTZ-0.5, in contrast with BTO, can adopt values of 〈*d*_ave_〉 larger than 330 pc/N in its *Amm*2 state because it can sustain larger fluctuations of its polarization. Moreover, unlike BaTiO_3_, BCTZ-0.5 is prevented from having piezoelectric coefficients smaller than 225 pC/N because the narrow temperature stability of its macroscopic *Amm*2 phase (about 30 K in BCTZ-0.5 versus 60 K in BTO, see Fig. 1a,b) prevents the orthorhombic symmetry from reaching lower temperatures where thermal fluctuations are restricted. Note that the inherent relation between a limited temperature range of stability of the orthorhombic phase and large piezoelectricity is further demonstrated in the Supplementary Note 2, where we also examined BCTZ-0.4. As a matter of fact, in this latter system, the range of stability of its orthorhombic state further contracted (about 15 K), which results in even larger piezoelectric coefficients. However, such range of stability in BCTZ-0.4 occurs for temperatures higher than 300 K, which is detrimental to generate high room-temperature electromechanical response.

**Figure 3:**
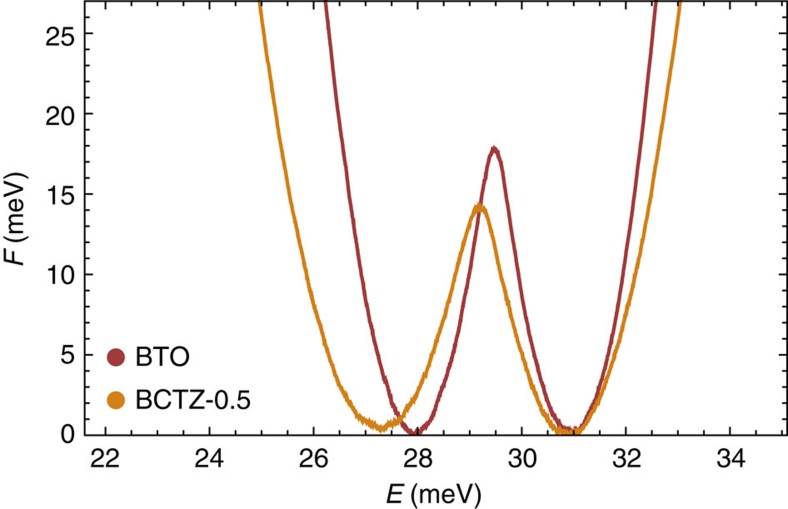
Free-energy landscape. Free-energy-like quantity versus internal energy (in meV per 5-atom cell) for (1−*x*)Ba(Zr_0.2_Ti_0.8_)O_3_−*x*(Ba_0.7_Ca_0.3_)TiO_3_ with *x*=0.50 (BCTZ-0.5) in yellow, and BaTiO_3_ (BTO) in red, at their *P*4*mm*–*Amm*2 transition temperatures (which are 297 and 283 K, respectively). Results correspond to the use of a 18 × 18 × 18 supercell. This free-energy-like quantity is the logarithm of the normalized canonical distribution^28^.

To understand the larger fluctuations occurring in the orthorhombic phase of BCTZ-0.5 with respect to the case of BaTiO_3_, Fig. 3 displays a quantity related to free energy–internal energy of both systems at their *P*4*mm*–*Amm*2 transition, as obtained using the Wang-Landau algorithm of ref. 28 within the effective Hamiltonians presently developed and/or used here. More precisely, this quantity related to free energy corresponds to the logarithm of the canonical probability function^28^. Figure 3 demonstrates the existence of two minima of similar free energy in both systems. The right minimum with larger internal energies corresponds to the macroscopic *P*4*mm* state while the left minimum with smaller internal energies is associated with *Amm*2. The existence of these two minima demonstrates the first-order character of the *P*4*mm*–*Amm*2 transition. Figure 3 also indicates that the energetic barrier between these two minima is smaller in BCTZ-0.5 than in BTO, therefore making the exploration of different orthorhombic states of different polarization direction, via an intermediate tetragonal state, easier of access close to the *P*4*mm*–*Amm*2 transition in (Ba_0.85_Ca_0.15_)(Zr_0.10_Ti_0.90_)O_3_ than in BaTiO_3_. This finding is fully consistent with the suggestion of ref. 11 that BCTZ-0.5 possesses a low energetic barrier between different ferroelectric states that allows its polarization to easily rotate and that results in large piezoelectric responses very near the *P*4*mm*–*Amm*2 transition. Furthermore and understand Figs 2a and 3 also reveals that the free energy–internal energy curve is much flatter around the orthorhombic minimum in BCTZ-0.5 than in BTO. In other words, there is a wider range of orthorhombic states having different internal energies (and thus different magnitudes of the polarization) that hold a similar free-energy in BCTZ-0.5. As a result, BCTZ-0.5 can exhibit larger fluctuations of its polarization within the macroscopic *Amm*2 phase. Note also that the existence of a low free-energy barrier and of flat minima revealed in Fig. 3 for BCTZ-0.5 is consistent with the proximity of a tricritical point in the phase diagram of (1−*x*)Ba(Zr_0.2_Ti_0.8_)O_3_−*x*(Ba_0.7_Ca_0.3_)TiO_3_, as discussed in ref. 11 (see Supplementary Note 2 for our predictions and discussion about tricritical point in this solid solution).

### Cluster analysis

Let us now determine whether the piezoelectric response and polarization’s fluctuations of BCTZ-0.5 and BTO correlate with microscopic features. For that, we identified clusters of tetragonal (T), Orthorhombic (O) and Rhombohedral (R) symmetry (within which dipoles nearly all lie along a 〈001〉, 〈110〉 and 〈111〉 pseudo-cubic direction, respectively) in both BCTZ-0.5 and BaTiO_3_, using a modified version of the Hoshen–Kopelman algorithm^29^,^30^. Interestingly, we numerically found (not shown here) that both systems support T, O and R clusters in their macroscopic *P*4*mm* state; this is reminiscent of the coexistence of *P*4*mm*, *Amm*2 and *R*3*m* phases close to the *P*4*mm*–*Amm*2 transition, reported in ref. 13 based on a Rietveld analysis of X-ray powder diffraction for a (Ba_0.85_Ca_0.15_)(Zr_0.10_Ti_0.90_)O_3_ sample. Moreover, while our T clusters fully vanish in the *Amm*2 state of these two materials, the O and R clusters remain, which bears resemblance with the decrease of the per cent of *P*4*mm* phase experimentally reported in refs 13, 14 when subjecting BCTZ-0.5 to electric field or stress near room temperature. These changes in microstructures are associated with the huge piezoelectric response found at the *P*4*mm*–*Amm*2 transition occurring between 296 and 298 K. Furthermore, our findings about local clusters demonstrate that the microscopic and macroscopic symmetries of BaTiO_3_, but also of BCTZ-0.5, can be quite different, which is in line with the celebrated Comes–Guinier–Lambert model^19^. On the other hand, our predictions of the existence of T clusters (in the *P*4*mm* phases) and O clusters (in both the *P*4*mm* and *Amm*2 phases) in addition to R clusters are in line with the findings of ref. 31 and go beyond the Comes–Guinier–Lambert model, since this latter model only expects R clusters (with different 〈111〉 directions) to occur in the macroscopic *P*4*mm* and *Amm*2 phases.

Let us focus on the macroscopic orthorhombic *Amm*2 phase of both BCTZ-0.5 and BTO since we are interested in relating its large piezoelectric coefficients displayed in Fig. 1c with atomistic characteristics. In this phase, we numerically found that the R clusters are dynamical in nature^32^, since they can change of location within the supercell and can also jump from one 〈111〉 direction to another 〈111〉 direction between different MC sweeps. Note that jump of polarization is typically associated with the so-called central mode, as demonstrated in ref. 33 for pure BaTiO_3_. On the other hand, we further discovered that there are two different types of O clusters in the macroscopic orthorhombic *Amm*2 phase of BCTZ-0.5 and BaTiO_3_. One type has a strong dynamical character as a result of the different 〈110〉 directions and locations within the supercell it can adopt during the MC simulations at fixed temperature. On the other hand, the second type of O clusters has a pronounced static character in the sense that its polarization is always oriented along the spontaneous polarization. However, this second type of cluster also possesses some dynamics, that is, it breathes rather than change location during these simulations, which may be related to the soft-mode that has been predicted and observed to exist (in addition to the central mode) in BTO^33^,^34^. As a result, such second type of O cluster can be referred to as quasi-static (it is worthwhile to realize that our simulations thus show that both quasi-static and dynamical clusters can coexist inside a pure system, such as BaTiO_3_, and not only in complex solid solutions such as relaxor ferroelectrics^35^). Interestingly, this quasi-static type of O cluster is, in fact, the so-called percolating cluster that spreads from one side of the supercell to its opposite side along the [100], [010] or [001] pseudo-cubic directions^36^.

**Figure 4:**
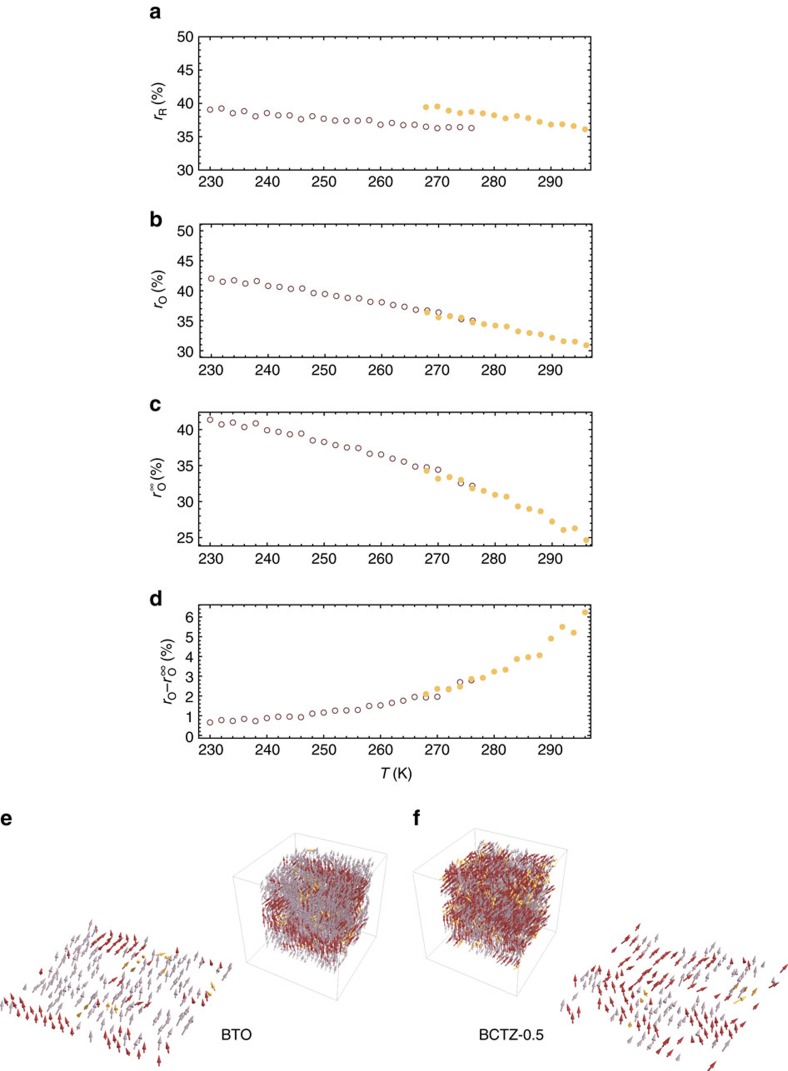
Cluster analysis. (**a**,**b**) Display the percentage occupied by the R and O clusters within the volume of the supercell (*r*_R_ and *r*_O_). (**c**) Represents the 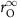 strength of the percolating O cluster (that is, the infinite cluster that spreads from one side of the supercell to its opposite side along the [100], [010] or [001] pseudo-cubic directions^36^). (**d**) Represents the difference between *r*_O_ and 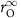. These data (**a**–**d**) are shown for BCTZ-0.5 (filled symbols) and BTO (open symbols) in their macroscopic *Amm*2 state, and correspond to the average over 100 different dipolar configurations (associated with 100 different MC sweeps) at any considered temperature *T* in a 18 × 18 × 18 supercell. (**e**,**f**) Display examples of R clusters (in red) as well as the two types of O clusters (the non-percolating in yellow, and percolating ones in purple) as occurring within the supercell and in a given *xy* supercell cross-section in BTO at 250 K and in BCTZ-0.5 at 280 K, respectively.

To corroborate these observations, we have conducted additional molecular dynamics simulations so as to estimate the relative time-scale of cluster dynamics in BCTZ-0.5 within the orthorhombic phase, at 280 K. We found that the polarization of the percolating O cluster does not change orientation throughout the 400 ps total simulation time, thus being indeed quasi-static at these time scales accessible to molecular dynamics simulations. Moreover, we found that the autocorrelation time of the volume of the non-percolating O clusters is of ∼1.4 ps, while for the percolating O cluster, it is of ∼2.3 ps, hence indicating that the latter, while featuring significantly slower dynamics nevertheless has a breathing component to its time evolution, essentially due to volume fluctuations. Examples of R clusters, as well as the two types of O clusters, are shown in Fig. 4e,f for both BCTZ-0.5 and BaTiO_3_. Figure 4a–d indicate the relative evolution with temperature of different types of clusters in BCTZ-0.5 and BaTiO_3_, and show that the priorly evidenced enhancement of piezoelectric response in the *Amm*2 phase of the former is associated with a more fragmented local structure. Specifically, Fig. 4a reports the ratio between the number of sites belonging to all R clusters over the total number of sites in the whole supercell in the macroscopic orthorhombic *Amm*2 phase of these two systems and as a function of temperature, while Fig. 4b displays the same ratio but for all O clusters. These two ratios are denoted as *r*_R_ and *r*_O_, respectively. Figure 4c shows the so-called strength of the percolating O cluster, 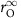, corresponding to the per cent of sites belonging to the (infinite) percolating O cluster. Furthermore, Fig. 4d reports the difference between *r*_O_ and 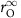, that is it represents the per cent of total volume occupied by the aforementioned first type of O clusters (that is, by the dynamical O clusters). Figure 4a,b demonstrate that the R and O clusters occupy a significant amount of the whole supercell in the macroscopic *Amm*2 state of both BCTZ-0.5 and BTO. For instance, for BaTiO_3_ at 240 K, *r*_R_ and *r*_O_ are both close to 40%. Interestingly, comparing Fig. 4b–d also tells us that most of the space occupied by the O clusters originates from the percolating O cluster, as demonstrated by the fact that *r*_O_–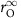 is always smaller than ≃6% for any temperature and decreases down to ≃1% when decreasing the temperature to 230 K. Other important information provided by Fig. 4 is that *r*_O_ and thus 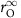 are rather sensitive to temperature in the *Amm*2 state of both BCTZ-0.5 and BTO, unlike *r*_R_. For instance, 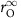 increases from 25 to 41% when decreasing temperature from 296 to 230 K, while *r*_R_ remains close to 40% in both the studied materials in that temperature range. Recalling that the range of stability of the *Amm*2 state typically occurs for higher temperatures in BCTZ-0.5 than in BaTiO_3_, one can therefore conclude that the local structure of the *Amm*2 phase of BCTZ-0.5 is more disordered/fragmented than that of BaTiO_3_. Such enhanced disordering allows for easier fluctuations of the polarization, and thus according to Fig. 2a to larger piezoelectricity. Figure 2b confirms the correlation between large piezoelectricity and enhancement of disordering of the local structure, as well as further sheds light into the strong connection between large electromechanical responses and percolating clusters, since 〈*d*_ave_〉 is found to typically increase when 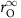 decreases in both BCTZ-0.5 and BTO.

To confirm this observation, we have additionally estimated the contribution to piezoelectricity stemming from each type of clusters in the *Amm*2 phase of BCTZ-0.5 (at 280 K) and BTO (at 250 K), by first determining at each MC sweep which local modes belong to which type of clusters and then using equation (2) for the local modes associated with each type of clusters. We found that, in the case of BCTZ-0.5 (BTO), the percolating O cluster, which occupies 31% (38.3%) of the supercell, has an individual piezoelectric response of 70.5 pC/N (48.9 pC/N), while the dynamical R and non-percolating O clusters, occupying, respectively, 38.4% (37.8%) and 3.3% (1.2%) of the supercell, have piezoelectric contributions of 68.8 pC/N (27.3 pC/N) and 29.5 pC/N (11.5 pC/N). These results consistently indicate that the larger the volume of the percolating O cluster, the lower is its contribution to the piezoelectric response (see Supplementary Note 1 for additional information about BCTZ-0.5). In light of these results, the trend line to achieve enhanced piezoelectricty appears to rest upon the relative fragmentation of local order. The latter can be tuned via *x* (see Supplementary Note 2 for additional information about BCTZ-*x*), via the application of a small electric field along one of the equivalent 〈111〉 directions that would depopulate the percolating O cluster, or alternatively, given the interplay between epitaxial strain and the orientation and morphology of local order^37^, via the application of epitaxial strain. Note, however, that these levers that would allow the tuning of the ratio of R and non-percolating O clusters to percolating O clusters in favour of the former ones are in interplay with temperature, a parameter that is intrinsically related to the studied phenomenon via thermal fluctuations.

## Discussion

In summary, atomistic simulations within an effective Hamiltonian scheme predict that BCTZ-0.5 undergoes a *P*4*mm*–*Amm*2 transition that occurs near room temperature, and that yields an orthorhombic state that has a rather flat free-energy landscape as well as a small temperature range of stability. A a result, larger fluctuations of the polarization occur in the *Amm*2 state of BCTZ-0.5 with respect to BaTiO_3_, thereby inducing higher piezoelectric responses near 300 K. Moreover, our study further reveals that this larger piezoelectric response is intrinsically linked to a specific feature of the local structure, namely the smaller strength of the percolating cluster. Interestingly, such cluster is of orthorhombic rather than rhombohedral local symmetry, and, as result, is missing in the famous Comes–Guinier–Lambert model^19^. In other words, such latter model ought to be generalized (by including O clusters in the *Amm*2 phase) be able to capture the microscopic origins of physical properties of BCTZ-0.5 and BTO.

Note that our study focuses on single phases. However, we also expect that the formation and coexistence of several phases inside BCTZ-0.5 will contribute to further enhancing piezoelectricity. This expectation stems from the fact that the barrier height of the free energy corresponds to the interface tension or, in other words, to the energy of the domain wall between phases of different symmetry^38^, and, from this perspective, the reduction of the barrier height revealed in Fig. 3 when going from BaTiO_3_ to BCTZ-0.5 enables domain wall fluctuations that should further strengthen the electromechanical response^39^.

We hope that such findings not only provide a better understanding of BCTZ-*x* systems but also can be used in the quest for other lead-free systems with high electromechanical conversion. For instance, our results suggest that one possibility for generating large piezoelectricity is to mix one system having a single-phase transition from cubic paraelectric to ferroelectric rhombohedral at a temperature to be denoted by *T*_c1_, with another material having another single-phase transition but from cubic paraelectric to ferroelectric tetragonal at a temperature to be denoted *T*_c2_. This mixing can then result in the emergence of a ferroelectric orthorhombic state having flat free-energy minimum, within a narrow temperature region that is located in-between the temperature stability regions of the ferroelectric tetragonal and ferroelectric rhombohedral states. The key factor to then obtain large room-temperature piezoelectric response is to make the stability of the orthorhombic state occurring in a region comprising room temperature, which can happen by finding the right amount of mixing between the two compounds and the right *T*_c1_ and *T*_c2_ temperatures. Note that, in this scenario, the existence of a tricritical point, where the tetragonal and rhombohedral ferroelectric states meet with the paraelectric phase, can occur at a specific composition and temperature but the highest room-temperature piezoelectric response can correspond to other compositions, namely the ones for which the orthorhombic state is stable within a small temperature region comprising 280–300 K. These are precisely the conditions encountered in BCTZ-*x* (refs 12, 23). Note also that this scenario is different from, that is, the mixing of rhombohedral Pb(Zr,Ti)O_3_ of lower Ti compositions with tetragonal Pb(Zr,Ti)O_3_ of larger Ti concentrations for which the coexistence of rhombohedral and tetragonal domains^40^, or the occurrence of a compositionally induced bridging monoclinic phase^4^,^15^, can yield large electromechanical response, since our scenario consists in creating a macroscopic orthorhombic phase of small temperature range of stability in-between the temperature ranges of the tetragonal and rhombohedral states.

## 

### Data availbility

The data that support the findings of this study are available from the corresponding author upon reasonable request.

## Additional information

**How to cite this article:** Nahas, Y. *et al*. Microscopic origins of the large piezoelectricity of leadfree (Ba,Ca)(Zr,Ti)O_3_. *Nat. Commun.* **8,** 15944 doi: 10.1038/ncomms15944 (2017).

**Publisher’s note**: Springer Nature remains neutral with regard to jurisdictional claims in published maps and institutional affiliations.

## Supplementary Material

Supplementary Information

Peer Review File
