# Corrigendum: Microscopic origins of the large piezoelectricity of leadfree (Ba,Ca)(Zr,Ti)O_3_

**DOI:** 10.1038/ncomms16172

**Published:** 2017-10-25

**Authors:** Yousra Nahas, Alireza Akbarzadeh, Sergei Prokhorenko, Sergey Prosandeev, Raymond Walter, Igor Kornev, Jorge Íñiguez, L Bellaiche

Nature Communications 8: Article number: 15944 ; DOI: 10.1038/ncomms15944 (2017); Published: 06 20 2017; Updated: 10 25 2017

The affiliation details for Sergei Prokhorenko are incorrect in this Article. A second affiliation should have been included as given below:

Theoretical Materials Physics, Q-MAT CESAM, Université de Liège, B-4000 Sart Tilman, Belgium.

Also, the financial support for this Article was not fully acknowledged. The Acknowledgements should have included the following:

Sergei Prokhorenko thank the University of Liege and the EU in the context of the FP7-PEOPLE-COFUND-BeIPDproject.

