# Supplementary material for: Microscopic origins of the large piezoelectricity of leadfree (Ba,Ca)(Zr,Ti)O3
Source: Nat Commun. 2017 Jun 20;8:15944. doi: 10.1038/ncomms15944 (PMC5481827; doi:10.1038/ncomms15944)
Supplement: Supplementary Information [file ncomms15944-s1.pdf]

Type of file: PDF
Size of file: 0 KB
Title of file for HTML: Supplementary Information
Description: Supplementary Figures, Supplementary Notes and Supplementary References

Type of file: PDF
Size of file: 0 KB
Title of file for HTML: Peer Review File
Description:

Supplementary Note 1: Additional information for BCTZ-0.5

Supplementary Figure 1 provides the temperature evolution of the elastic coefficients versus temperature in BTO and in BCTZ-0.5, as predicted by the effective Hamiltonian approach described in the manuscript and based on the virtual crystal approximation (VCA) [1, 2]. We compute $(C_{11} + 2C_{12})/3$, with C_{11} and C_{12} being elastic coefficients (with the indices referring to the basis formed by the pseudo-cubic [100], [010] and [001] directions) and being practically calculated by using the fluctuation-dissipation theorem – which involves correlations of strain tensor components. Figure S1 predicts a pronounced decrease of elastic coefficients in BCTZ-0.5 near the $Pm\bar{3}m$–$P4mm$, $P4mm$–$Amm2$ and $Amm2$–$R3m$ phase transitions, which is consistent with the elastic softening of the lattice observed in Ref. [3] and suggested in Ref. [4].

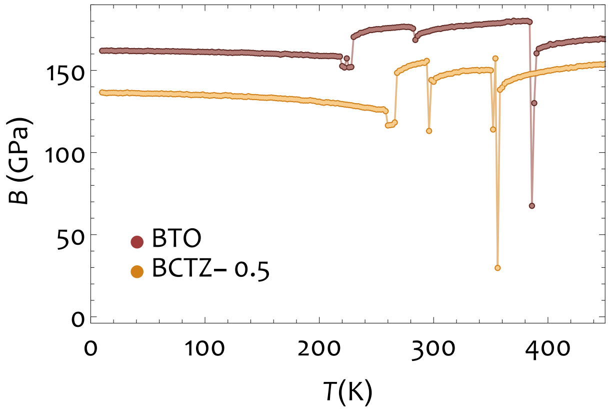

Supplementary Figure 1: **Elastic coefficients.** Temperature evolution of the elastic coefficients in BTO and in $(1-x)Ba(Zr_{0.2}Ti_{0.8})O_3$–$x(Ba_{0.7}Ca_{0.3})TiO_3$ with $x = 0.50$, as predicted within our VCA-based effective Hamiltonian and using a $18 \times 18 \times 18$ supercell.

Furthermore, Supplementary Figure 2 reports the predicted temperature dependence of one third of the trace of the dielectric tensor of BCTZ-0.5, as computed from a correlation function involving fluctuations of the supercell average of the local modes [5, 6] and as obtained when using our VCA-based effective Hamiltonian approach. This dielectric component is enhanced near the phase transitions, especially around the paralectric cubic-to-ferroelectric tetragonal one as also observed in Refs. [3, 4]. One can also realize that our computed dielectric response can be larger than 3,000 around room temperature as also found in Ref. [4], which further emphasizes the validity of the VCA-based approach to model BCTZ systems.

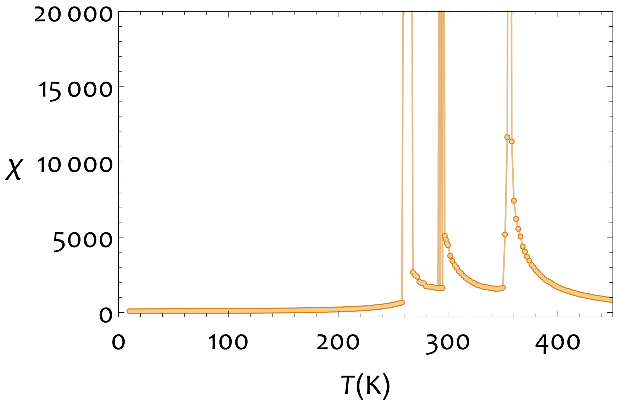

Supplementary Figure 2: **Dielectric susceptibility.** Temperature dependence of one third of the trace of the dielectric tensor in BCTZ-0.5, as predicted within our VCA-based effective Hamiltonian and using a $18 \times 18 \times 18$ supercell. Note that this dielectric component reaches values that are larger than 20,000 very near the $Pm\bar{3}m$–$P4mm$, $P4mm$–$Amm2$ and $Amm2$–$R3m$ transitions and that are not shown here.

We have also computed, using Supplementary Eq. 2, the contributions of each type of clusters, namely, R clusters,

percolating O cluster (static), and non-percolating O clusters (dynamic), to the dielectric responses in the case of BCTZ-0.5 at 280 K, i.e., within the O-phase, where they occupy 38.4%, 31%, and 3.3% of the supercell, respectively. We found that the largest contribution stems from R clusters (551.5), followed by the percolating O cluster (296.3), and the non-percolating O clusters (165.3). These results show that, although the R clusters and the percolating O cluster occupy comparable volumes in the supercell, the contribution of the R cluster is almost 2 times bigger than that of the percolating cluster, due to the dynamic behavior of the former. Similarly, despite the non-percolating clusters having almost ten times smaller volume compared to that occupied by the percolating O cluster, their contribution to the dielectric response is only twice lower, compensation brought by their dynamic behavior. Note however that the full susceptibility also includes term stemming from correlations of polarization between different cluster types that we have not computed.

Moreover, Supplementary Figure 3 shows the temperature evolution of the Cartesian components of the supercell average of the local modes (such average is directly related to the spontaneous polarization) for BCTZ-0.5 when cooling down versus heating this system. One can see that the cooling and heating procedures provide very similar results, including the value of the critical temperatures of the $Pm\bar{3}m$–$P4mm$, $P4mm$–$Amm2$ and $Amm2$–$R3m$ phase transitions. Such findings therefore reveal rather small temperature hysteresis of these transitions, as consistent with the measurements of Ref. [4] and the fact that the energetic barriers separating phases near these transitions are rather small (see Fig. 3 of the manuscript).

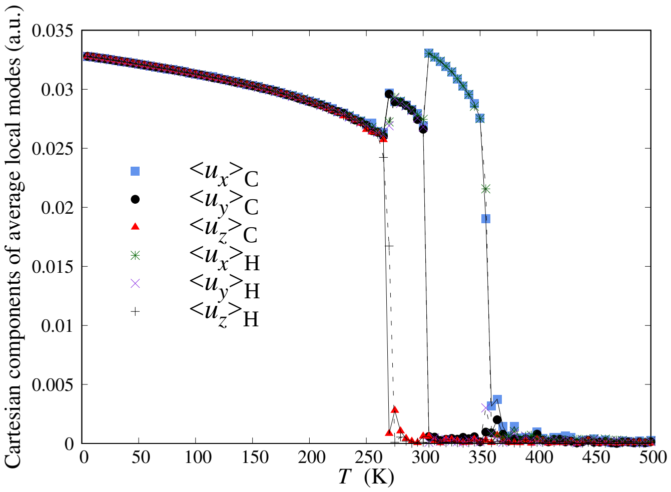

Supplementary Figure 3: **Evolution of spontaneous polarization upon cooling and heating.** Temperature dependence of the absolute values of the Cartesian components of the supercell average of the local modes in BCTZ-0.5 under cooling and heating, as obtained from the use of a 12 × 12 × 12 supercell. The 'C' and 'H' subscripts are used in the legend to indicate the results from the cooling and heating procedures, respectively

Supplementary Note 2: Additional information for BCTZ-x

Let us also demonstrate that effective Hamiltonian (H_{eff}) schemes based on the virtual crystal approximation (VCA) [1, 2] can accurately describe the temperature versus composition phase diagram of $(1-x)Ba(Zr_{0.2}Ti_{0.8})O_3$–$x(Ba_{0.7}Ca_{0.3})TiO_3$ (BCTZ-x) in some concentration range encapsulating x=0.50, including non-trivial features.

To this end, in addition to the H_{eff} built for x=0.50 indicated in the manuscript, we also built effective Hamiltonians for $(1-x)Ba(Zr_{0.2}Ti_{0.8})O_3$–$x(Ba_{0.7}Ca_{0.3})TiO_3$ solid solutions, with x varying between 0.25 and 0.65 while still using the same analytical expression of the total energy as in Ref. [7, 8]. To do that, we further employed the virtual crystal approximation [1, 2] for the composition of x=0.40. As for the concentration of x=0.50 discussed in the manuscript, the parameters of the effective Hamiltonian for x=0.40 were extracted by performing density functional (DFT) calculations within the VCA approach [2], then modifying the κ_2 and γ coefficients of Eq. (1) of the manuscript for $(1-x)Ba(Zr_{0.2}Ti_{0.8})O_3$–$x(Ba_{0.7}Ca_{0.3})TiO_3$ alloys with x=0.40 to reproduce measured Curie temperatures [4, 9, 10] as well as the lowest transition temperature [4, 9] for this composition. All the effective Hamiltonian parameters for any concentration x varying between 0.25 and 0.65 were then obtained by linearly interpolating or extrapolating those

of the two compositions of 0.40 and 0.50. Supplementary Figure 4 shows the resulting predicted temperature versus composition phase diagram, as obtained from analyzing the polarization.

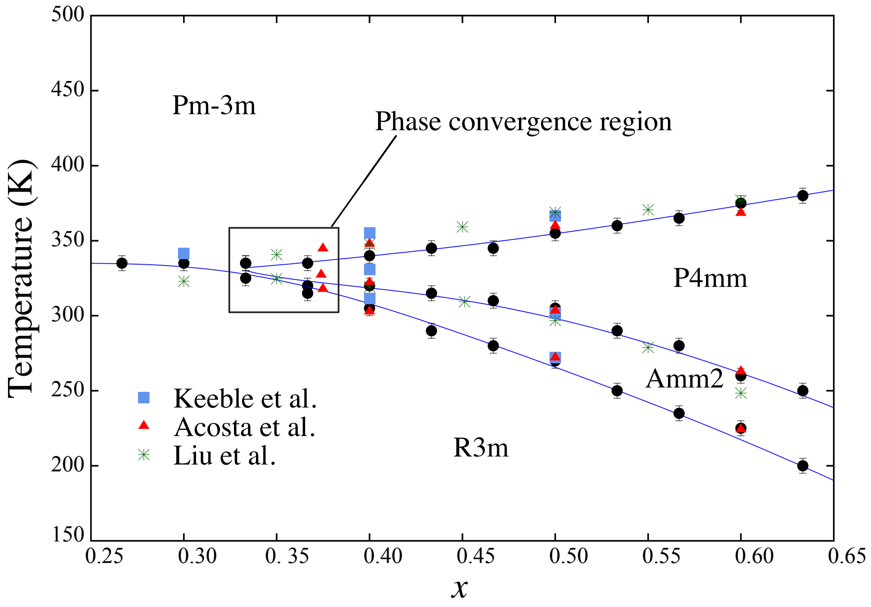

Supplementary Figure 4: **Phase diagram.** Temperature versus composition phase diagram of $(1-x)\mathrm{Ba(Zr_{0.2}Ti_{0.8})O_3}$–$x\mathrm{(Ba_{0.7}Ca_{0.3})TiO_3}$ for x varying between 0.25 and 0.65. The black symbols display our predicted computational results, while experimental data of Refs. [4, 9, 10] are shown by means of blue, red and green symbols, respectively. Solid lines are guide for the eyes. Technically, the computational data correspond to Monte-Carlo simulations using 100,000 sweeps and 12 × 12 × 12 supercells, and transition temperatures are determined based on the temperature behavior of the spontaneous polarization.

One can see that the computational results agree well qualitatively but also quantitatively with the experimental data of Refs. [4, 9, 10], further attesting the validity of our approach. In particular, the calculations correctly predict three successive transitions (these are $Pm\bar{3}m$–$P4mm$, $P4mm$–$Amm2$ and $Amm2$–$R3m$) when decreasing the temperature for x above 0.37, as in Refs. [9, 10] (note that Ref. [4] seems to have overlooked the existence of an orthorhombic state in $(1-x)\mathrm{Ba(Zr_{0.2}Ti_{0.8})O_3}$–$x\mathrm{(Ba_{0.7}Ca_{0.3})TiO_3}$). Furthermore, our calculations yield a phase convergence region around x=0.33 and T =330 K for which the ferroelectric rhombohedral $R3m$, orthorhombic $Amm2$ and tetragonal $P4mm$ phases, as well as the cubic $Pm\bar{3}m$ phase, should all have close free energies. Moreover, we find a single transition from cubic paraelectric $Pm\bar{3}m$ to ferroelectric rhombohedral $R3m$ for x equal or below 0.3, similarly to Refs. [4, 9, 10].

Note that the aforementioned rescaling of the κ_2 and γ parameters is needed to have a phase diagram that quantitatively agrees with measurements. For instance, using the initial values of $\kappa_2 = 0.0626$ a.u. and $\gamma = 0.4453$ a.u. (along with a negative pressure of -4.8 GPa, as done in Ref. [7, 8] for pure $\mathrm{BaTiO_3}$) provides for x=0.50 critical temperatures of $\simeq$ 127 K, 121 K and 116 K for the cubic-to-tetragonal, tetragonal–to-orthorhombic and orthorhombic-to-rhombohedral transitions, respectively. They are therefore smaller and closer to each other than the corresponding values of $\simeq$ 360 K, 297 K and 270 K obtained when adopting the presently renormalized $\kappa_2 = 0.0383$ a.u. and $\gamma = 0.4186$ a.u. (without applying a negative pressure). The need for such rescaling, in general, and the reduction of κ_2, in particular (which results in deeper energy wells), may originate from our use of the local approximation within DFT to extract the parameters of the effective Hamiltonian, as argued in a recent work [11]. It may also be due to the virtual crystal approximation by itself.

In order to assess whether the piezoelectric properties of BCTZ-0.5 are affected by the proximity of a tricritical point (as suggested in Ref. [4]) and/or depend on the small temperature range of stability of the $Amm2$ state (as we additionally advocate in the manuscript), we undertook further examination of the properties of an intermediate composition, namely $(1-x)\mathrm{Ba(Zr_{0.2}Ti_{0.8})O_3}$–$x\mathrm{(Ba_{0.7}Ca_{0.3})TiO_3}$ with $x = 0.40$. In panels (a) and (b) of Supple-

mentary Figure 5, we show the supercell average of the local modes and the average $\langle d_{ave}\rangle$ piezoelectric coefficient. We find that this composition features an $Amm2$ phase with even narrower range of stability (around 15 K versus 30 K for BCTZ-0.5 and 60 K for BTO_3) and higher $P4mm$-to-$Amm2$ transition temperature (around 315 K). The corresponding piezoelectric response is found to reach values ranging between $\simeq$ 250 and 550 pC/N, compared with the values ranging between $\simeq$ 225 pC/N and 525 pC/N in the $Amm2$ phase of BCTZ-0.5. In light of these additional results, the trend upon approaching the tricritical point is the simultaneous contraction of the $Amm2$ phase and elevation of the $P4mm$-to-$Amm2$ transition to higher temperatures. Such latter features allows greater thermal fluctuations and therefore enable an enhancement of piezoelectricity but at temperatures higher than 300 K. In this regard, $(1-x)\mathrm{Ba(Zr_{0.2}Ti_{0.8})O_3}$–$x\mathrm{(Ba_{0.7}Ca_{0.3})TiO_3}$ with $x = 0.50$ is an optimal composition for technological applications in that the large piezoelectric response, although not extremal, ideally occurs near room temperature.

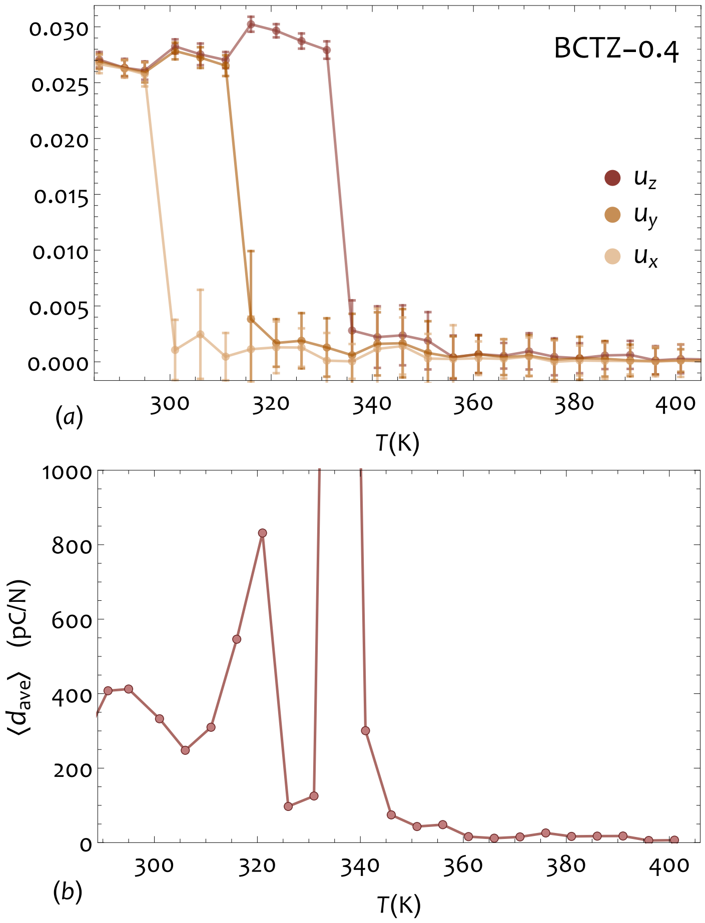

Supplementary Figure 5: **Macroscopic properties.** Temperature dependence of properties of $(1-x)\mathrm{Ba(Zr_{0.2}Ti_{0.8})O_3}$–$x\mathrm{(Ba_{0.7}Ca_{0.3})TiO_3}$ with $x = 0.40$, as obtained from the use of 18 × 18 × 18 supercells. Panel (a) reports the supercell average of the local modes (in lattice constant units), while panel (b) displays the average $\langle d_{ave}\rangle$ piezoelectric coefficient (see manuscript for the definition of $\langle d_{ave}\rangle$). Note that $\langle d_{ave}\rangle$ reaches values larger than 1,000 at the paraelectric-to-ferroelectric transition. Such values are not shown here because they will make challenging to see piezoelectricity of the order of hundreds of pC/N.

Supplementary Note 3: Relationship between piezoelectricity and fluctuations of polarization

Let us start by adapting the relationship given in, e.g,. Ref. [12] that expresses the proportionality between the d_{ii} piezoelectric coefficient (when using Voigt notation) and the product of the χ_{ii} element of the dielectric tensor and the i-component of the local mode as averaged over the supercell and MC sweeps at any chosen temperature, $u_{s,i}$:

$$d_{ii} = B\ \chi_{ii}\ u_{s,i} \tag{1}$$

where B is a coefficient that is directly proportional to electrostrictive coefficients (which are typically independent of temperature [13]). Note that $u_{s,i}$ is directly related to the i-component of the spontaneous polarization and that the ratio between piezoelectric coefficient and the product of spontaneous polarization with a dielectric component is known to be rather constant and independent of temperature in various ferroelectrics, for instance as in experimental results for $LiTaO_3$ [14], as consistent with Supplementary Eq. (1).

It is also known that χ_{ii} can be expressed as [5, 6] :

$$\chi_{ii} = \frac{C}{T}\{\langle u_i \; u_i\rangle - \langle u_i\rangle\langle u_i\rangle\}, \tag{2}$$

where T is temperature, u_i is the i-component of the supercell average of the local mode at a given MC sweep, and $\langle \; \rangle$ denotes statistical averages (over the different MC sweeps). Moreover, $C = \frac{(N_s Z^*)^2}{V\varepsilon_0 k_{\rm B}}$, where N_s is the number of the 5-atom cells in the supercell, Z^* is the dynamical charge of the local mode, V is the volume of the supercell, ε_0 is the vacuum dielectric permittivity, and $k_{\rm B}$ is the Boltzmann constant.

Let us now express u_i as:

$$u_i = u_{s,i} + \delta u_i \tag{3}$$

where δu_i is the fluctuation of u_i with respect to $u_{s,i}$ during the MC sweeps.

Plugging Supplementary Eq. (3) into Supplementary Eq. (2), and imposing that $\langle \delta u_i\rangle = 0$ (since this fluctuation can be equally positive or negative) yields:

$$\chi_{ii} = \frac{C}{T}\langle \delta^2 u_i\rangle \tag{4}$$

As a result, Supplementary Eq. (1) becomes:

$$d_{ii} = B \; C\frac{u_{s,i}}{T} \; \langle \delta^2 u_i\rangle \tag{5}$$

Finally, let us recall that the average $\langle d_{ave}\rangle$ coefficient of the manuscript is defined as $\frac{d_{11}+d_{22}+d_{33}}{2}$ in the *Amm2* phase, with the 1, 2 and 3 indices referring to the pseudo-cubic [100], [010] and [001] directions, respectively. Moreover, when choosing the polarization of this latter phase to lie along [011], one has $u_{s,1} = 0$ and $u_{s,2} = u_{s,3} = \frac{u_s}{\sqrt{2}}$, where u_s is the magnitude of the local mode as averaged over the supercell and MC sweeps (u_s is directly proportional to the spontaneous polarization). As a result, Supplementary Eq. (5) leads to:

$$\langle d_{ave}\rangle = \frac{B \; C}{\sqrt{2}}\frac{u_s}{T}\{\langle \delta^2 u_2\rangle + \langle \delta^2 u_3\rangle\} \tag{6}$$

Once knowing that we numerically found that $\frac{u_s}{T}$ is nearly independent of the temperature in the macroscopic *Amm*2 phase of both BCTZ-0.5 and $BaTiO_3$, Supplementary Eq. (6) explains the linear relationship depicted in Fig. 2a of the manuscript.

Supplementary References

[1] Van Vechten, J.A. Quantum dielectric theory of electronegativity in covalent systems. I. Electronic dielectric constant. *Phys. Rev. A* **182**, 891-905f (1969).

[2] Bellaiche, L. & Vanderbilt, D. Virtual crystal approximation revisited: Application to dielectric and piezoelectric properties of perovskites. Phys. Rev. B **61**, 7877 (2000).

[3] Acosta, M., Khakpash, N., Someya, T., Novak, N., Jo, W., Nagata, H., Rossetti, G. & Rödel, J. Origin of the large piezoelectric activity in $(1-x)$Ba$(Zr_{0.2}Ti_{0.8})O_3$–$x$$(Ba_{0.7}Ca_{0.3})TiO_3$ ceramics. Phys. Rev. B **91**, 104108 (2015).

[4] Liu, W. & Ren, Z. Large Piezoelectric Effect in Pb-Free Ceramics. Phys. Rev. Lett. **103**, 257602 (2009).

[5] Bin-Omran, S., Kornev, I.A., & Bellaiche, L. Wang-Landau Monte Carlo formalism applied to ferroelectrics. Phys. Rev. B **93**, 014104 (2016).

[6] García, A. & Vanderbilt, D. in *First-Principles Calculations for Ferroelectrics: Fifth Williamsburg Workshop*, (Ed. Cohen, R. E.) 53-60 (AIP, New York, 1998)

[7] Zhong, W., Vanderbilt, D. & Rabe, K. M. Phase transition in $BaTiO_3$ from first principles. Phys. Rev. Lett. **73**, 1861 (1994)

[8] Zhong, W., Vanderbilt, D. & Rabe, K. M. First-Principles theory of ferroelectric phase transitions for perovskites: the case of $BaTiO_3$. Phys. Rev. B **52**, 6301 (1995).

[9] Keeble, D.S., Benabdallah, F. , Thomas, P.A, Maglione, M. & Kreisel, J. Revised structural phase diagram of $Ba(Zr_{0.2}Ti_{0.8})O_3$–$(Ba_{0.7}Ca_{0.3})TiO_3$. Appl. Phys. Lett. **102**, 092903 (2013).

[10] Brandt, D.R., Acosta, M., Koruza, J. & Webber, K.G. Mechanical constitutive behavior and exceptional blocking force of lead-free BZT-xBCT piezoceramics. J. Appl. Phys. **115**, 204107 (2014).

[11] Paul, A., Sun, J., Perdew, J.P. & Waghmare U.V. Accuracy of first-principles interatomic interactions and predictions of ferroelectric transitions in perovskite oxides: Energy functional and effective Hamiltonian. Phys. Rev. B **95**, 054111 (2017).

[12] Kvasov, A. & Tagantsev, A.K. Role of high-order electromechanical coupling terms in thermodynamics of ferroelectric thin films. Phys. Rev. B **87** 184101 (2013).

[13] Kuwata, K., Uchino K. and Nomura S. Electrostrictive Coefficients of $Pb(Mg_{1/3}Nb_{2/3})O_3$ Ceramics. Jpn. J. Appl. Phys. **19**, 2099 (1980).

[14] Lines, M.E. & Glass A.M. Principles and Application of Ferroelectrics and Related Materials (Clarendon Press, Oxford 1977).
